# Enhancing the Data Reliability of Multilevel Storage in Phase Change Memory with 2T2R Cell Structure

**DOI:** 10.3390/mi12091085

**Published:** 2021-09-09

**Authors:** Yi Lv, Qian Wang, Houpeng Chen, Chenchen Xie, Shenglan Ni, Xi Li, Zhitang Song

**Affiliations:** 1 State Key Laboratory of Functional Materials for Informatics, Laboratory of Nanotechnology, Shanghai Institute of Microsystem and Information Technology, Chinese Academy of Sciences, Shanghai 200050, China; lvyi@mail.sim.ac.cn (Y.L.); qian.wang@mail.sim.ac.cn (Q.W.); xcc@mail.sim.ac.cn (C.X.); nisl@mail.sim.ac.cn (S.N.); ituluck@mail.sim.ac.cn (X.L.); ztsong@mail.sim.ac.cn (Z.S.); 2 University of Chinese Academy of Sciences, Beijing 100049, China; 3 Shanghai Technology Development and Entrepreneurship Platform for Neuromorphic and AI SoC, Shanghai 200090, China

**Keywords:** phase change memory, resistance drift, 2T2R, multilevel storage

## Abstract

Multilevel storage and the continuing scaling down of technology have significantly improved the storage density of phase change memory, but have also brought about a challenge, in that data reliability can degrade due to the resistance drift. To ensure data reliability, many read and write operation technologies have been proposed. However, they only mitigate the influence on data through read and write operations after resistance drift occurs. In this paper, we consider the working principle of multilevel storage for PCM and present a novel 2T2R structure circuit to increase the storage density and reduce the influence of resistance drift fundamentally. To realize 3-bit per cell storage, a wide range of resistances were selected as different states of phase change memory. Then, we proposed a 4:3 compressing encoding scheme to transform the output data into binary data states. Therefore, the designed 2T2R was proven to have optimized storage density and data reliability by monitoring the conductance distribution at four time points (1 ms, 1 s, 6 h, 12 h) in 4000 devices. Simulation results showed that the resistance drift of our proposed 2T2R structure can significantly improve the storage density of multilevel storage and increase the data reliability of phase change memory.

## 1. Introduction

With the popularization of electronic equipment, the position of memory in the semiconductor market is growing. In accordance with Moore’s law, devices are decreasing in size. The charge-based memories, such as DRAM and flash, having dominated the market for decades, are reaching their physical limits in scaling. This has motivated research on next-generation memories [1].

At present, research on memory is mainly on high density, high speed and reliability. Phase change memory (PCM) has attracted wide attention in the semiconductor field due to its advantages of high erasure times [2], simple structure, good compatibility with CMOS process, low cost and wide range of resistances [3,4,5].

Multilevel storage technology is an effective way to improve storage density and reduce data storage costs. The remarkable difference in electrical properties between the crystalline and amorphous states of phase change materials makes the intermediate resistance state of PCM have great value for utilization. It is suitable for multivalued storage technology. At the same time, with the advent of the era of artificial intelligence, MLC PCM is expected to constitute a computing system integrating storage and operation. However, the current data reliability of multilevel storage in PCM technology still faces the restriction of resistance drift [6,7]. Resistance drift is a thermally-affected phenomenon; it increases the resistance over time [8,9]. Unlike single level storage, there may be overlaps between adjacent resistance states over time, resulting in some data errors due to the narrow state interval of each resistance state in multilevel storage.

Numerous approaches to tackle the resistance drift phenomena have been developed, some through materials engineering. For example, Rao. F et al. designed a phase-change heterostructure (PCH) that consists of alternately stacked phase-change and confinement nanolayers to suppress the noise and drift [10]. Others through electronic means. Various architectural methods have been introduced to improve the reliability of multilevel cell (MLC) PCM. A sensing concept accommodated eight resistance levels in three in-dependent 10X sensing windows to tolerate resistance drift without closing the memory windows was introduced by J. Y. Wu et al. [11]. W. -S. Khwa et al. proposed a resistance drift compensation scheme for MLC PCM to mitigate against R-drift without such compromises [12]. M. Imran et al. put forward an MLC PCM architecture based on effective data encoding with a simple flip operation applied to the data prior to a write to enhance the reliability of MLC PCM [13]. Different encoding techniques have also been proposed which can considerably enhance the reliability. T. Kwon presented an XOR-based encoding scheme to mitigate the resistance drift of the MLC PCM [14]. A pattern redistribution technique according to the rate of resistance drift for different storage levels proportioned the data-patterns to improve the MLC PCM reliability was discussed by M. Imran et al. [15]. However, these architectures and encoding methods cannot completely eliminate the influence of resistance drift, and requires an error correction scheme, which brings a large number of additional parity bits to store resulting in reduction of storage density. Current pulses for MLC PCM programming and the delay of long encoding and decoding for error correction codes will significantly reduce performance and energy efficiency.

Based on the above, by analyzing the overlapping problem caused by resistance drift, this paper proposes a method of multilevel storage with 2T2R structure to enhance the data reliability. Meanwhile, the influence of increasing circuit area caused by the addition of error correction circuit is avoided. In this paper, we introduce a 2T2R structure for multilevel storage in phase change memory to enhance the data reliability. Based on the characteristic of PCM and resistance drift on multilevel storage in PCM, the proposed 2T2R structure adopts the combination of 2 PCM cells with wide range resistance. Moreover, 4:3 compressing encoding scheme is demonstrated to transform the state code to binary code realizing 3 bit/cell storage. Eventually, Conductance drift of proposed 2T2R structure achieved 8 states shows the high data reliability with time growing.

The rest of this paper is organized as follows. In Section 2, PCM technology and multilevel storage are demonstrated. Correspondingly, the 2T2R structure of multilevel storage design is explored in phase change memory and readout and write operations in Section 3. Section 4 presents the results of experiments with the proposed structure. Comparison and analysis of the designed circuits are provided in Section 5. Finally, Section 6 presents the conclusions drawn from this work.

## 2. Phase Change Memory Theory

### 2.1. Background of Phase Change Memory

Nonvolatile phase-change random-access memory (PCM) is regarded as a leading candidate for next-generation electronic memory hierarchy [10]. Phase change memory is an element based on Ovshinsky effect, which changes the resistance difference between crystalline and amorphous of phase change materials, so as to realize the writing and erasing information [16]. Phase change materials can not only be converted between crystalline and amorphous states, but also realize a variety of different states with obvious differences from complete crystal state and amorphous state under different certain programming pulses [17]. The resistances of these intermediate states also correspond with the resistances of completely crystalline state and completely amorphous state. This characteristic of phase change materials can be used to realize multilevel storage of PCM.

**Figure 1:**
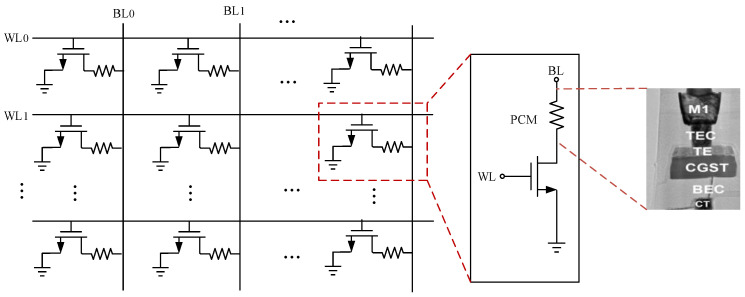
A circuit schematic of a memory cell with a phase change element.

**Figure 2:**
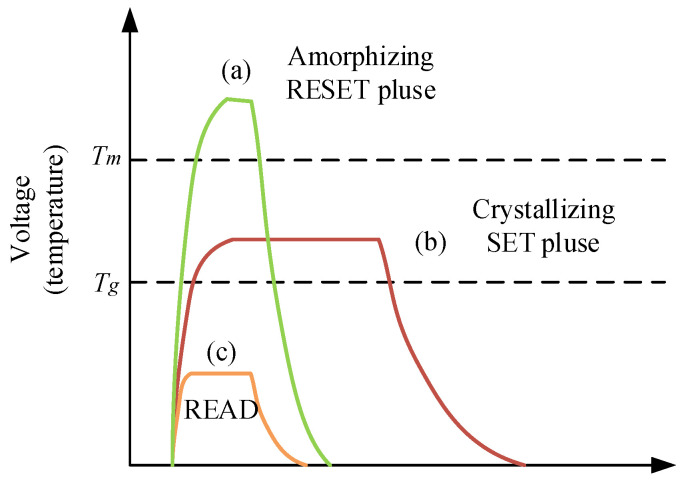
An illustration of RESET, SET and READ programming pulses.

Figure 1 introduces the circuit schematic of a memory cell with a phase change element. An illustration of RESET, SET and READ programming pulses for PCM is shown in Figure 2. By applying a short and high pulse as shown in (a) to the memory cell, the temperature of the phase change material is heated above the melting point, and then the temperature drops suddenly to maintain the high resistance amorphous transformation, and the reset operation is completed to realize the storage of data “0.” The long and medium amplitude pulse shown in curve (b) is applied to the memory cell to heat the phase change material temperature above the crystallization point and below the melting point, so as to complete the crystal transformation of low resistance, achieving the set operation and the storage of data “1.” The short and weak pulse shown in (c) is used to read out the resistance state of PCM without changing the state of phase change material.

### 2.2. Multilevel Storage of Phase Change Memory

The multilevel characteristic of phase change resistance is usually used for phase change memory multilevel storage; that is, phase change resistance can be programmed to an intermediate resistance state, between high resistance and low resistance, to realize the storage of multiple bit in a single cell. However, due to the deviations of process conditions, the resistance value of phase change memory is not a certain value but a certain distribution.

**Figure 3:**
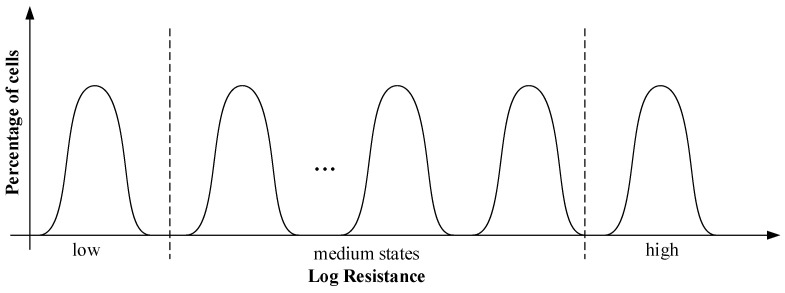
Resistance distribution of multilevel storage in phase change memory.

Figure 3 is the resistance distribution of phase change memory during multilevel storage. In multilevel storage, the resistance drift in the intermediate state will cause the unit to represent the wrong data; that is, when reading data, the initial resistance may exceed the predetermined range after drift and overlap with the adjacent resistance. Moreover, the resistance drift will cause the changed resistance state to become wider with the elapsed time, thereby limiting the number of intermediate states for reliable programming. (N−2) resistance range (as shown in the medium state in the Figure 3) should be inserted in the gap between the high and low resistance states to realize the storage of N states. However, the gap between the adjacent resistance ranges will become narrower, which will have a great impact: on the one hand, the sensitivity of the readout amplifier should be high enough to distinguish the small resistance value difference; on the other hand, the error of misreading will occur when the process fluctuation causes the overlap of the adjacent resistance range, which will greatly affect the reliability of the PCM.

**Figure 4:**
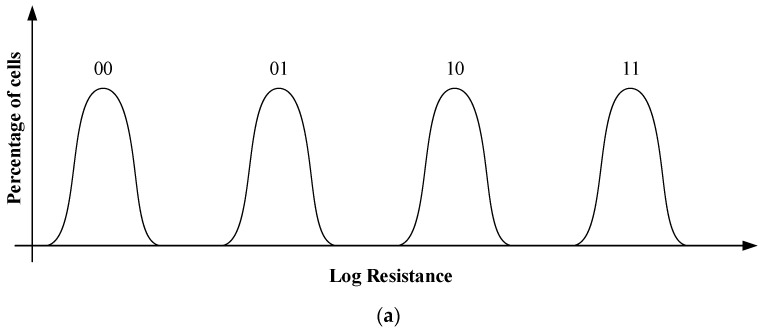

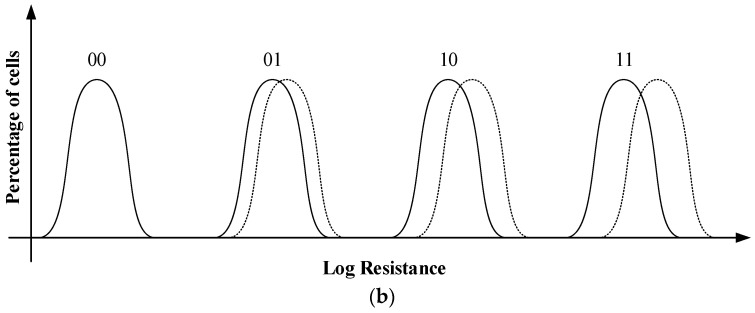
(**a**) Ideal resistance distribution. (**b**) Resistance distribution in reality.

Due to the characteristics of phase change materials, the resistance of PCM cell will change with time, resulting in resistance drift [18]. In the multilevel storage of PCM, the resistance values of intermediate states overlap with those of other adjacent resistance states, which leads to readout errors. Figure 4a shows the resistance distribution of 2-bit storage on PCM. From the Figure 4b, the curves are drifting by the decrease in the resistance.

At present, the most widely used cell structures of PCM are 1T1R and 2T2R. 1T1R is widely used in multilevel storage because of its high storage density and simple structure, but it is easily affected by process fluctuations, which leads to the misreading of data. In contrast, 2T2R shows obvious advantages in anti-interference of process fluctuation and reliable information storage.

## 3. Multilevel Phase Change Memory

### 3.1. 2T2R Structure

**Figure 5:**
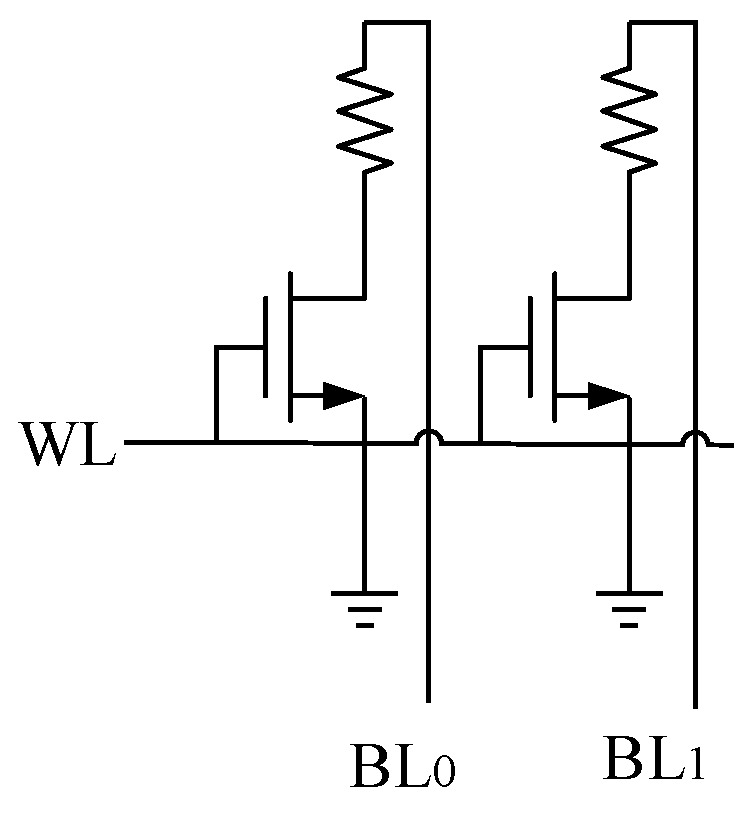
2T2R structure of PCM for multilevel storage.

In order to reduce the complexity of sensitive amplifier design and improve the reliability of high-density multilevel storage, 2T2R structure is used in this design for multilevel storage in PCM, as shown in Figure 5. Each PCM has at least three or more stable resistive states. Through the different resistance combinations of two PCM cells, eight states with large differences in resistances were selected, and eight data states were selected for multilevel storage. The three stable resistive states in PCM cells from small to large are called resistive state L, resistive state M, and resistive state H.

**Figure 6:**
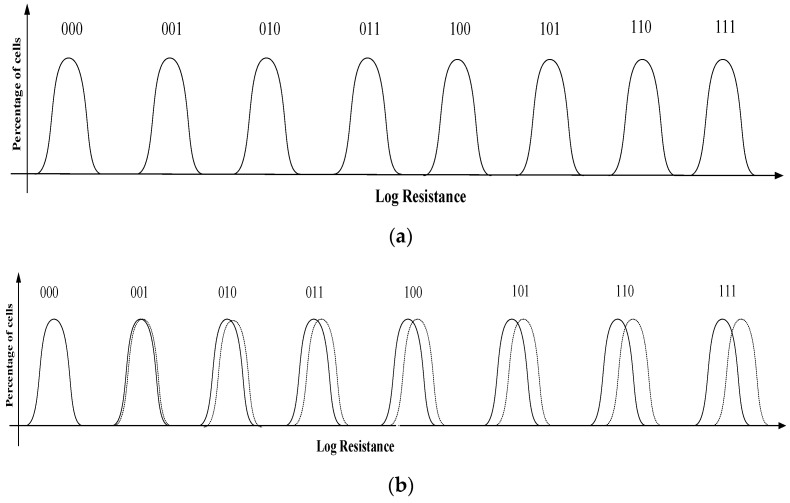
(**a**) The 8-state resistance distribution of PCM with the proposed 2T2R structure. (**b**) Actual resistance distribution of PCM with the proposed 2T2R.

Figure 6 shows the 8-state resistance distribution curve of PCM and the actual distribution under the influence of resistance drift. The curves “000,” “001,” …and “111” correspond to the data of two PCM in different resistance states. According to the introduction of background technology, PCM will experience a resistance drift phenomenon over time. It can be seen from Figure 6b that the resistance will increase with the time of storage. After a long period of time, there may be overlaps among the resistance states, which will lead to misreading in the data reading process, reducing the reliability of PCM.

As the resistance range of PCM is wide, different resistance states are realized through the resistance combination of two units to avoid the overlap of resistance states. The corresponding relationship between the resistance state combination of the two PCM resistance states and the binary data states is shown in Table 1. In order to ensure the symmetry of code distance, “MM” state is eliminated here.

### 3.2. Multilevel Storage System

PCM can display different resistance states under different program pulses. The difference in resistance from the fully crystalline state to the completely amorphous state can be up to four orders of magnitude. By operating the storage cells in several states with large difference in resistance, multilevel storage of 3-bit or even more bits of data in each PCM cell can be realized.

**Figure 7:**
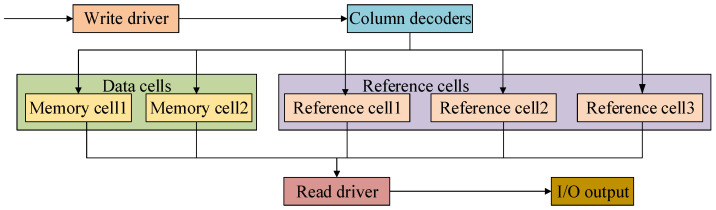
A schematic of the multilevel phase change memory system.

In the multilevel PCM system proposed in the paper, shown in Figure 7, two PCM cells in each 2T2R PCM unit have three or more stable resistance states through different writing and erasing pulses.

**Figure 8:**
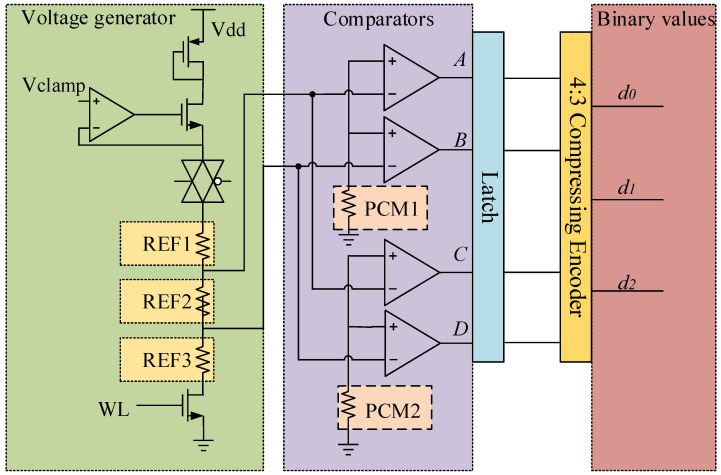
A schematic of the comparison readout circuit.

The paper provides a structure of the readout circuit in Figure 8. The first component is a voltage generator composed with a resistor ladder of high, medium and low resistances set from top to bottom that provides voltage references, which are divided into equally space values, for an output. The three reference cells generate two reference voltages for comparison with the voltage of the data cell in the next step. The second component is a set of comparators. Each comparator compares input voltage of PCM data cells and reference voltage. Output of a comparator is digital value 1 if input voltage is higher than reference voltage. Output of a comparator is digital value 0 if input voltage is lower than reference voltage. The third component is a latch for data storage. The outputs of comparators have the form of a state code. The last component is a compressing encoder converting input state code to output binary code.

When reading out the data from PCM cells, the resistance of PCM is not convenient for judging directly; we need to change it into current or voltage. The schematic of readout is shown in Figure 8. Select memory cell 1 and memory cell 2 of data units through column decoders and transmit the state of data unit to output comparison readout circuit.

### 3.3. 4:3 Compressing Encoder

As the resistance is not easy to be measured directly, the method of readout voltage is adopted in this design. By setting the reference unit, the information stored in the data unit can be read out correctly. From the comparator, we can conclude the data “00” represents low resistance, “01” represents medium resistance and “11” represents high resistance. Through the mapping Table 2, it shows 8 different states and realizes 3 bit information storage. The resistance of the reference units will also change along with the resistance drift, so the readout process can be regarded as unaffected. With time going by, the resistance of reference unit also changes. We can easily get the resistance code.

Here, we specify that the read-out data of the circuit is labeled A, B, C, D from left to right and the data states are marked $d_{0}$, $d_{1}$, $d_{2}$. We can get the logical expression from the Table 2 as follows:

(1) $$d_{0}=\bar{A}BCD+AB\bar{CD}+AB\bar{C}D+ABCD,$$

(2) $$d_{1}=\bar{A}\bar{B}CD+\bar{A}B\bar{CD}+AB\bar{C}D+ABCD,$$

(3) $$d_{2}=\bar{A}\bar{B}\bar{C}D+\bar{A}B\bar{CD}+AB\bar{CD}+ABCD,$$

Through simplification, we obtain the function

(4) $$HSB=d_{0}=BD+A,$$

(5) $$MSB=d_{1}=\bar{A}B\bar{D}+AD+\bar{B}C,$$

(6) $$LSB=d_{2}=\bar{B}\bar{C}D+B\bar{D}+AC,$$

**Figure 9:**
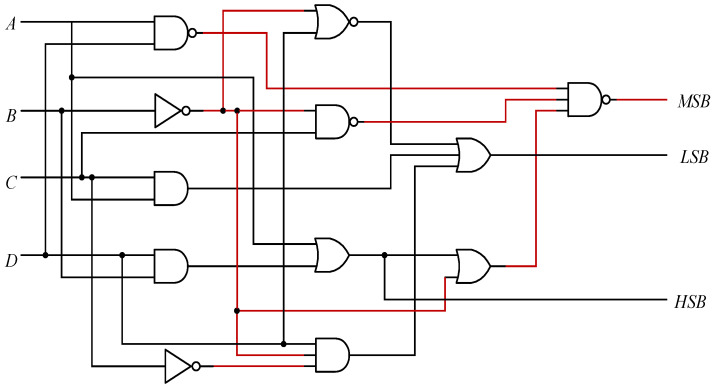
A schematic of the encoder circuit.

The encoder circuit shown in Figure 9 can be obtained according to the above truth Table 2.

## 4. Experimental Results

**Figure 10:**
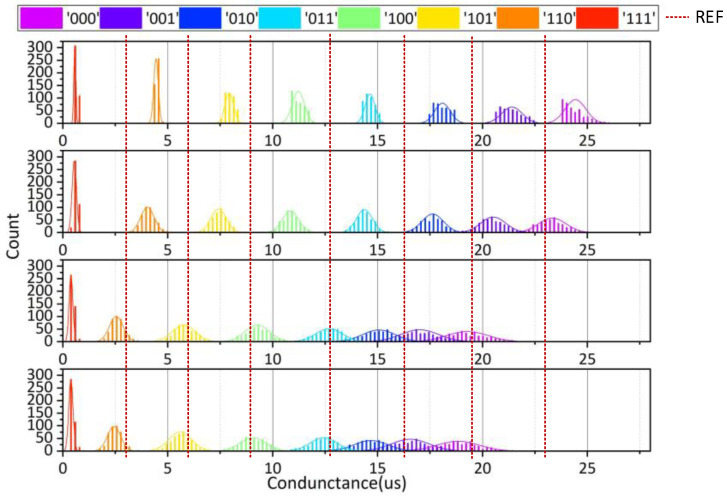
The conductance drift of the conventional 1T1R structure in 8 states.

**Figure 11:**
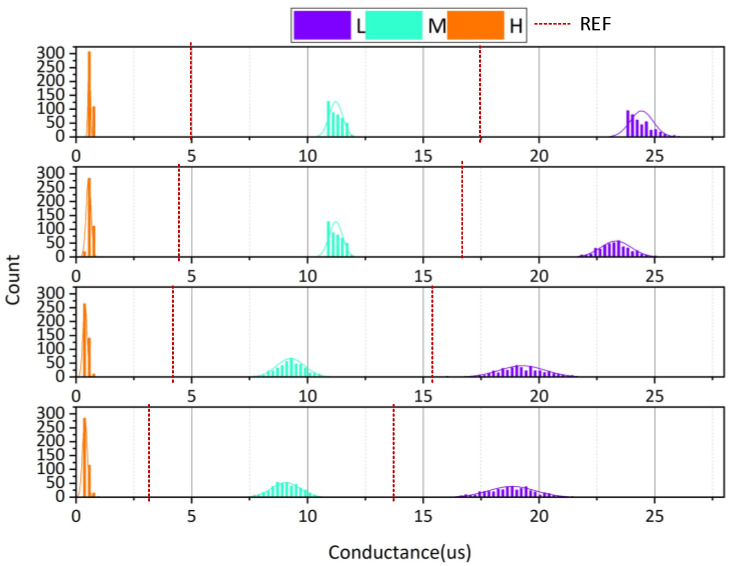
The conductance drift of the proposed 2T2R structure in 8 states.

To evaluate the proposed 2T2R structure, we demonstrate the conductance distribution at four time points (1 ms, 1 s, 6 h, 12 h) from 4000 devices with the conventional 1T1R and the proposed 2T2R structures, shown in Figure 10 and Figure 11, respectively.

With the traditional 1T1R structure, the method of bisection is usually used to read the storage cell. For the reference cell, we usually take half of the maximum resistance and the minimum resistance. When the resistance of storage cell is larger than the reference cell, data “1” is output, and when the storage cell is smaller than the reference cell, data “0” is output. It could be noticed that the conductance of each state becomes lower, resulting in more overlap between adjacent states.

Compared with the conductance drift distribution diagram of conventional 1T1R structure, the separation of each state using the proposed 2T2R structure is good enough to distinguish eight states, although the resistance still increased slightly with time.

**Figure 12:**
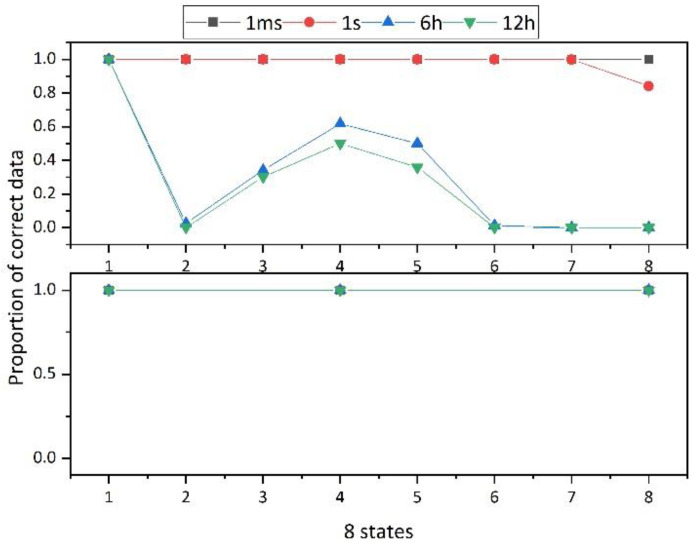
Comparison of the likelihood of reading the correct data between conventional 1T1R and the proposed 2T2R structure.

The 2T2R structure proposed in this paper selects three resistance values with large differences for multilevel storage, as presented in Figure 11. When reading data, the same phase change memory is used as the reference cell, and the resistance value of the reference unit is between the resistance values of the selected resistance values. As time elapses, the resistance of the reference cell and the resistance of the data cell will change at the same time, which reduces the impact of drift and ensures the reliability of data. The probabilities of reading the correct data after resistance drift in 1T1R and 2T2R structures are shown in Figure 12. It is obvious from the figure that 2T2R is more robust with time.

## 5. Comparison

In this section, we present the performance of the proposed 2T2R structure compared with that of the conventional MLC PCM based on 1T1R and 2T1R structures [19,20] in Table 3.

1T1R structure consists of one transistor and one PCM. The area of 1T1R is low but it can only store 1 bit. The traditional 1T1R structure is to operate the memory cell by adding different voltages to bit line. The structure is simple and occupies a small area, but the storage density is low. Meanwhile, a reliability problem will follow because of the uncertainty of the resistance of PCM affected by the drift.

1T2R structure consists of one transistor and two PCM. The area of 1T2R is larger than that of 1T1R. The traditional 1T1R structure can only reduce the size and the operating current of PCM. Two cells can be separately accessed by individual bit line. In case memory density is fixed, the transistor of 1T2R structure can be larger than that of 1TIR structure while having higher current driving capability. Although the density is improved, there are still some problems with the reliability of the data.

The 2T2R structure consists of two transistors and two PCM. The proposed 2T2R structure can store more bits for large applications. While the storage capacity is doubled, the area is increased by only 1/5. The designed reference unit cannot only reduce the resistance drift but also improve the accuracy of data storage. Our work can expand storage capacity efficiently and greatly increase the reliability of information storage.

## 6. Conclusions

This paper provides a solution to multilevel storage in PCM adopted the structure of 2T2R as the basic cell for data storage, which realize the storage of 3bit or more data per cell through the combination of different resistance values of 2 PCM elements, reducing the influence of resistance drift and realizing the high-density storage of PCM. 4:3 compressing encoder is proposed for obtain binary data values. The simulation results show good performance of the feasibility on the proposed structure and the data reliability of multilevel storage in PCM. Eventually, a 2T2R structure at 3 bit/cell showing distinct distribution is more attractive because of its advantages in storage density and reliability, and the design complexity of circuit.

## Figures and Tables

**Table 1:** The relationship of data states and PCM resistance states.

2-PCM Resistance States	Binary Data
LL	000
LM	001
LH	010
ML	011
MH	100
HL	101
HM	110
HH	111

**Table 2:** The mapping relationship between readout states and data states.

2-PCM Resistance States	Readout States	Binary Data
LL	0000	000
LM	0001	001
LH	0011	010
ML	0100	011
MH	0111	100
HL	1100	101
HM	1101	110
HH	1111	111

**Table 3:** The comparison between the proposed structure and the previous.

PCM Structure			
Reference	[19]	[20]	This paper
Circuit schematic	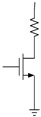	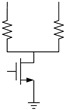	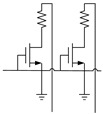
Structure	1T1R	1T2R	2T2R
Area	24	40	48
Data states	2	4	8
Data capacity	low	low	high
Storage density	1 bit/cell	2 bit/cell	3 bit/cell
Data reliability	low	average	high

## Data Availability

Not Applicable.
